# A BAYESIAN NETWORK-BASED APPROACH FOR MULTI-OMICS INTEGRATION TO REVEAL UNDERLYING MECHANISMS OF HEALTHY AGING

**DOI:** 10.1093/geroni/igad104.2477

**Published:** 2023-12-21

**Authors:** Anastasia Leshchyk, Stefano Monti, Paola Sebastiani

**Affiliations:** Boston University, Boston, Massachusetts, United States; Boston University, Boston, Massachusetts, United States; Tufts Medical Center, Boston, Massachusetts, United States

## Abstract

Previous research on long-lived individuals showed that centenarians significantly delay the onset of the disability and aging-related diseases such as Alzheimer’s, dementia, and cardiovascular diseases to the very end of their lives compared to the general population. Genetic studies of centenarians and healthy agers showed that carriers of the APOE e2 allele had increased odds of reaching longevity compared to the non-e2 allele carriers. In addition, the APOE e2 allele is characterized by distinct serum proteomics and metabolomics profiles that could be useful to understand the mechanism of propagation of the genetic effect of APOE to the molecular level and eventually to phenotypes. We are developing a novel network-based approach of multi-layer data integration to identify shared molecular profiles among the subjects with familial longevity that lead to compression of morbidity, disability, and mortality. This Bayesian network-based approach integrates proteomics, metabolomics, genetics, and multiple phenotype data to decipher the risk factors and pathways contributing to prolonged health span. For example, the preliminary analysis shows that the centenarians with cognitive performance above the average in their age and sex group have lower levels of IL6, GDF15, and PTN proteins than those with below-average cognitive performance. These known inflammation factors might impair the cognitive function of elderly adults contributing to their disability and morbidity, thus affecting their aging.

